# Remarks on Cataract; with a Case

**Published:** 1846-05

**Authors:** William M. Boling

**Affiliations:** Montgomery, Alabama


ART. II.—*Remarks on Cataract; with a Case.* By WILLIAM M. BOLING, M.D., of Montgomery, Alabama.

The propriety of operating for cataract on the eye first affected, the other eye as yet remaining sound, has been a mooted question. The weight of the authority, in times past, has been decidedly against such a proceeding; recently it has been more equally balanced, and perhaps at the present time inclines somewhat in its favor.

The arguments against an early operation have been principally founded on facts or assertions like the following:—First, that one eye is amply sufficient for usual vision; consequently the patient's condition is not i.nproved by the operation, and he therefore undergoes the pain, and incurs the risk, without an adequate object. Second, that double vision and confusion in the appearance of objects arise from the difference in the foci of the two eyes, caused by the removal of the lens from one only.

It is strange that such objections should be allowed to have such weight, and it is surprising to see the latter especially, which may be so easily removed by properly adapted convex glasses, assigned by learned authors.

In regard to the former; although a very useful degree of vision may be enjoyed with one eye only, he is "*rara avis in terris*" indeed, who could think, after a successful operation which had restored to him the use of the other, that his condition was not improved, and that he had "incurred a risk without an object."

The principal ground on which an early operation is urged is, that it will probably prevent the formation of a cataract in the other eye. In support of this, cases are recorded by the best authorities, in which the spontaneous removal of an incipient opacity of the crystalline lens has taken place, on the removal of a cataract from the other eye. This is accounted for by the peculiar and intimate sympathy existing between the eyes, by which the sound one frequently takes on diseased action similar to that existing in the one first affected. Wardrop makes mention of a peculiar inflammatory affection which takes place in the eye of horses, producing disorganization of the internal structure, and states the fact, that its progress in the eye last affected is arrested by its removal from the one primarily diseased. In this country, the practice is not uncommon with farmers, under like circumstances, to remove the disorganized eye when the other becomes 'weak.'

It may be enquired, however, whether the predisposition to cataract manifested by its formation in the first eye, and the continuation of other causes which may have led to it, are not in many instances the cause also of its development in the second? This is a natural inquiry, and facts in all probability give it a positive answer. Under such circumstances, the removal of the first cataract would exert no control over the existing predisposition, and would probably not prevent, ultimately, the development of a second; still as some of the causes leading to such a result would thereby be removed, its progress, we may suppose, would be less rapid.

Even where no peculiar predisposition to cataract might be thought to exist, and without the intervention of this intimate sympathy between the eyes, the mere occurrence of blindness of one eye, from any other cause whatever, by throwing the labor of vision entirely on the other, entailing thus upon it a double amount of duty, may probably in some cases lead to the development of cataract in it. Supposing this to be true, it may also be urged, among other reasons, for the early removal of cataract existing in one eye only. The following case is one in which cataract was probably caused by the increased exertion of the organ, rendered necessary by the previous loss of vision in the other. There is no reason to suppose that any predisposition to cataract existed, or it would have been produced by the violence sustained by the eye first affected; while the agency of sympathy between the eyes in its production is rendered improbable, by the dissimilarity in the character of the affections of the two organs.

Case.—Nat. Newman, mulatto, aged 89 years, received twenty years ago a blow in his right eye with an ear of corn. Pain and inflammation followed, which in a short time were removed; but at the end of three years from the time of receiving the injury, the eye was entirely blind. Shortly after the entire loss of vision in the right eye, the sight of the left began to fail, and in two years more was also entirely lost. On the 20th of August last his eyes presented the following appearance. The pupil of the right eye was clear, of natural size, but almost entirely immovable. The amaurosis was complete; with this eye he had not the slightest perception of light. Of the left eye, the lens was opaque, the cataract being of a perfectly white color. With this eye he had a slight perception of light—sufficient, when in a dark room, on a bright day, with one window open, to enable him to discover its position. The conjunctiva of the eyes were somewhat inflamed, and the tarsi considerably so, and also thickened. By the removal of a few inverted cilia, which seemed to be measurably the cause of the conjunctival disease, and the use, night and morning, of mercurial ointment, with creosote, and one or two applications of nitrate of silver, this condition was in a great measure removed.

On the 4th of September I couched the cataract of the left eye, and his vision immediately after was such, that he could distinguish faces, and without difficulty could tell any number of fingers held up before him.

The ultimate termination of the case was unfortunate. The childish delight he felt in consequence of the restoration of the long lost function, caused him to exert and expose the organ more than was prudent; nor were admonitions to a contrary course availing. A high degree of inflammation was thus excited, which his age prevented me from treating with the activity necessary for its removal. Secondary cataract and eventually almost entire disorganization of the eye followed.

March, 1846.

